# Engineering of a Fully Human Anti-MUC-16 Antibody and Evaluation as a PET Imaging Agent

**DOI:** 10.3390/pharmaceutics14122824

**Published:** 2022-12-16

**Authors:** Hanan Babeker, Jessica Pougoue Ketchemen, Arunkumar Annan Sudarsan, Samitha Andrahennadi, Anjong Florence Tikum, Anand Krishnan Nambisan, Humphrey Fonge, Maruti Uppalapati

**Affiliations:** 1 Department of Pathology and Laboratory Medicine, College of Medicine, University of Saskatchewan, Saskatoon, SK S7N 5E5, Canada; 2 Department of Medical Imaging, College of Medicine, University of Saskatchewan, Saskatoon, SK S7N 0W8, Canada; 3 Department of Medical Imaging, Royal University Hospital Saskatoon, Saskatoon, SK S7N 0W8, Canada

**Keywords:** theranostics, immuno-PET/CT, fully human monoclonal antibody, MUC16, epithelial ovarian cancer, pancreatic ductal adenocarcinoma

## Abstract

Antibodies that recognize cancer biomarkers, such as MUC16, can be used as vehicles to deliver contrast agents (imaging) or cytotoxic payloads (therapy) to the site of tumors. MUC16 is overexpressed in 80% of epithelial ovarian cancer (EOC) and 65% of pancreatic ductal adenocarcinomas (PDAC), where effective ‘theranostic’ probes are much needed. This work aims to develop fully human antibodies against MUC16 and evaluate them as potential immuno-PET imaging probes for detecting ovarian and pancreatic cancers. We developed a fully human monoclonal antibody, M16Ab, against MUC16 using phage display. M16Ab was conjugated with p-SCN-Bn-DFO and radiolabeled with ^89^Zr. ^89^Zr-DFO-M16Ab was then evaluated for binding specificity and affinity using flow cytometry. In vivo evaluation of ^89^Zr-DFO-M16Ab was performed by microPET/CT imaging at different time points at 24–120 h post injection (p.i.) and ex vivo biodistribution studies in mice bearing MUC16-expressing OVCAR3, SKOV3 (ovarian) and SW1990 (pancreatic) xenografts. ^89^Zr-DFO-M16Ab bound specifically to MUC16-expressing cancer cells with an EC_50_ of 10nM. ^89^Zr-DFO-M16Ab was stable in serum and showed specific uptake and retention in tumor xenografts even after 120 h p.i. (microPET/CT) with tumor-to-blood ratios > 43 for the SW1990 xenograft. Specific tumor uptake was observed for SW1990/OVCAR3 xenografts but not in MUC16-negative SKOV3 xenografts. Pharmacokinetic study shows a relatively short distribution (t_1/2α_) and elimination half-life (t_1/2ß_) of 4.4 h and 99 h, respectively. In summary, ^89^Zr-DFO-M16Ab is an effective non-invasive imaging probe for ovarian and pancreatic cancers and shows promise for further development of theranostic radiopharmaceuticals.

## 1. Introduction

Ovarian and pancreatic cancers are among the cancers with the highest mortality rates. The current standard of care for epithelial ovarian cancer (EOC) is surgery and platinum-based chemotherapy [1]. However, nearly 85% of patients relapse with poor prognosis and develop resistance to further chemotherapy [2]. Pancreatic ductal adenocarcinoma (PDAC) has a 5-year survival rate of less than 5% and a 1-year survival of just under 25% [3]. Surgical resection followed by chemotherapy or radiotherapy improves overall survival (OS) [4]. However, most patients are diagnosed with advanced disease with very poor outcomes. Therefore, there is an urgent need for effective therapeutics and diagnostics for early detection of both ovarian and pancreatic cancers.

Aberrant overexpression of cell-surface receptors in cancer cells can be targeted by monoclonal antibody drugs. In addition, these antibodies can be used as a carrier to deliver contrast agents (for imaging) and/or cytotoxic payloads (for therapy) directly to the site of tumors. There is immense interest in developing antibody-based radiopharmaceuticals using a “theranostics” approach where the same molecule can be used both for non-invasive imaging and targeted therapy. In this study, we seek to develop radiopharmaceuticals targeting the overexpression of biomarker, MUC16 (CA125), which is commonly overexpressed in ~80% of EOC and ~65% of PDAC.

MUC16 belongs to the mucin family of proteins. Mucins are high-molecular weight glycoproteins (>10^6^ Da) that are typically expressed on the apical surface of epithelial cells to serve as a protective barrier against stress and infection [5,6]. Some members of the family, such as MUC16 and MUC4, are membrane-anchored with short cytoplasmic tails thereby modulating signaling cascades in addition to their barrier functions [7]. Epithelial cancers are known to overexpress aberrant forms of mucins that promote survival, metastasis, and immune evasion [8]. Elevation of CA125/MUC16 in serum is detected in ~80% of epithelial ovarian cancers. It is a well-known biomarker for monitoring the progression and regression of disease [9,10]. MUC16 is a frequently mutated gene [11] and is associated with increased growth and metastasis [12,13]. The interaction of MUC16 with mesothelin leads to peritoneal metastasis of ovarian cancer cells by facilitating the attachment of cancer cells to the mesothelial lining [12,14]. Overexpression of MUC16 correlates with a worse prognosis in EOC [14,15,16,17]. MUC16 is not expressed in normal pancreatic ducts but is highly expressed in PDAC and associated metastatic lesions [18].

Several MUC16 antibodies of murine origin have been generated previously and some of these antibodies have been humanized for development as antibody-drug conjugates (ADCs) or as immuno-PET imaging probes [19,20,21,22,23]. Sharma et al. [24] reported the development of a ^89^Zr-labeled PET probe using a murine antibody B43.13 (oregovomab), while Olson et al. [25] reported the evaluation of a murine antibody AR9.6 labeled with IRDye800CW for image-guided surgery. To the best of our knowledge, there are no literature reports of a fully human anti-MUC16 antibody imaging probe. Here, we report for the first time the development of a fully human monoclonal anti-MUC16 antibody for the purpose of PET imaging and targeted radioimmunotherapy of MUC16-expressing cancers. We have developed and evaluated the ^89^Zr-labeled human antibody in murine models of MUC16-positive EOC and PDAC using microPET/CT and ex vivo biodistribution. Promising results, obtained in this study, lay the foundation for the future development of anti-MUC16-based radiopharmaceuticals with more potent radionuclides for targeted therapy.

## 2. Materials and Methods

### 2.1. Cloning of Domains SEA11-12 of MUC16

mRNA was isolated from OVCAR3 cells (obtained from ATCC) using an mRNA isolation kit (Roche Diagnostics GmbH, Mannheim, Germany) according to the manufacturer’s instructions. The cDNA was synthesized from 500 ng mRNA using Maxima First Strand cDNA Synthesis Kit for RT-PCR, with dsDNase (Thermo Fisher Scientific, Waltham, MA, USA), following the manufacturer’s instructions. The predicted DNA sequence of the SEA11-12 domain of MUC16 was obtained from the NCBI (GenBank Accession #: AF414442 and NM_024690). The following primers were used to amplify the coding sequence with overhangs for Gibson assembly into a custom vector pMUFV01-Fc.

HB103 (5′ to 3′)
GCATTGCACTAAGTCTTGCACTTGTCACGAATTCGATAAATGGTTTCACCCAGCGG
HB104 (5′ to 3′)
GGCATGTGTGAGTTTTGTCAGATCTAACCATGGCCGATGATAAATTCTGGGGTGCATAGC

This vector (Supplementary Figure S1) is a lentiviral compatible vector that contains a strong EF alpha promoter and encodes proteins with IL2 secretion signal and C-terminal Fc fusion.

### 2.2. Expression and Purification of Recombinant SEA11-12-Fc Protein

The recombinant protein for SEA11-12 Fc fusion was expressed and purified using transient transfection of sequence-verified plasmids in Expi293F suspension cells (Gibco). Expi293F cells were cultured using Expi293F complete media (Gibco). A total of 75 × 10^6^ Expi293F cells were transfected with 30 μg of SEA11-12 Fc DNA at 37 °C. Cells were harvested and pelleted after five days, and recombinant protein was purified from the supernatant using affinity chromatography (MabSelectSure resin, GE Healthcare) following manufacturer-recommended protocol. The integrity and purity of the protein were confirmed using SDS-PAGE.

### 2.3. Screening Naïve Antibody Libraries Using Phage Display

We used a naïve antibody library previously developed in our lab [26]. This library was panned against the recombinant SEA11-12 Fc fusion protein using previously described protocols [27]. Three rounds of selection were performed, and the selection pool was tested for specific binding to SEA11-12 protein. Clonal ELISA was performed using 24 clones isolated from the round 3 pool to test for specific binding to the target protein following standard protocols [27]. Clones specific to target recombinant SEA 11-12 domains were identified by DNA sequencing.

### 2.4. Expression of M16Ab Antibody in IgG1 Format

For the expression of lead anti-MUC16 antibody, M16Ab, in IgG1 format, the VL and VH coding sequences were cloned into a pFUSE2ss-CLIg-hK vector and pFUSE2ss-CHIg-hG1 vectors, respectively (Invivogen). Expi293F cells were used for secreted expression of full-length MUC16 IgG following previously described protocols [28]. M16Ab was purified using MabSelectSure (GE Healthcare) affinity resin using manufacturer-recommended protocols. Bioanalyzer (Agilent 2100 Bioanalyzer using Agilent High Sensitivity Protein 230 Kit) was used to analyze the purity of the protein following the manufacturer’s protocol.

### 2.5. Cell Lines and Mouse Xenograft Models

Human ovarian (OVCAR3; SKOV3) and pancreatic (SW1990) cancer cell lines were purchased from ATCC (Rockville, MD, USA). OVCAR3 cells were cultured in 1X RPMI medium (HyClone Laboratories, Logan, UT, USA), supplemented with 20% fetal bovine serum (FBS) (Biochrom) and 1% bovine insulin. SW1990 cells were propagated using Leibovitz’s L-15 medium (HyClone Laboratories, Logan, UT, USA) supplemented with 10% FBS. SKOV3 cells were cultured using McCoy medium (HyClone Laboratories, Logan, UT, USA) supplemented with 10% FBS. Both OVCAR3 and SKOV3 cells were incubated at 37 °C in a humidified atmosphere of 5% CO_2_, while SW1990 cells did not require CO_2_. Female NOD-SCID and CD-1 nude mice of 4 weeks of age were obtained from Charles River Canada (St-Constant, QC, Canada). At five weeks of age, NOD-SCID mice were subcutaneously co-injected with both 7 × 10^6^ SW1990 and 20 × 10^6^ OVCAR3 at the left and right hind limb, respectively, and separate NOD-SCID mice were injected with 10 × 10^6^ SKOV3 cells. All cells were prepared in 100 μL suspension of a 1:1 mixture of medium and Matrigel matrix basement membrane (Discovery Labware, Inc. Bedford, MA, USA). Only for OVCAR3 xenografts, a second injection was performed at the same site using 15 × 10^6^ OVCAR3 cells. CD-1 nude mice were injected with 7 × 10^6^ SW1990 cells in 100 μL suspension of a 1:1 mixture of medium and Matrigel matrix basement membrane. Tumor growth was measured using a digital caliper.

### 2.6. Flow Cytometry

In a 96-well non-binding plate, different concentrations of the M16Ab IgG diluted in 1X PBS buffer (300 nM, 100 nM, and 3 nM) were added to OVCAR3, SW1990, and SKOV3 cells seeded at 3 × 10^5^ cells/well. M16Ab was allowed to bind for 30 min at 4 °C. Cells were washed and re-suspended in ice-cold 1X PBS twice. Goat anti-Human IgG PE-conjugated secondary antibody (eBioscience, cat. #12-4998-82) was added to cells in (1 in 500) dilution and allowed to bind for 30 min at 4 °C. Cells were washed three times with ice-cold 1X PBS. To verify the expression of MUC16 in both cell lines, the commercial anti-MUC16 antibody X75 (Invitrogen) was used as a positive control at a concentration of 30 and 10 nM. The plate was read using a CytoFLEX machine (Beckman Coulter, add location) on the FL1 channel and flow cytometry data were analyzed using FlowJo version 10.6. The EC_50_ and K_D_ were determined using GraphPad Prism software.

### 2.7. Antibody Internalization

Incucyte live cell imaging S3 (Essen BioScience, Ann Arbor, MI, USA) was used to study the internalization of the DFO-M16Ab in MUC16-expressing OVCAR3 and SW1990 cells and MUC-16-negative SKOV3 cells as previously reported [ref]. Following live-cell imaging, the mean red object area (μm^2^/well) was calculated using the IncuCyte software, which is used to quantify the internalization uptake of the antibody [29].

### 2.8. Conjugation of Anti-MUC-16 Ab with p-SCN-Bz-Deferoxamine

Conjugation of the antibody to deferoxamine (*p*-SCN-DFO) for radiolabeling with ^89^Zr was carried out using standard lab SOPs [29]. The purity of M16Ab and DFO-M16Ab was determined using size exclusion HPLC (SEC-HPLC Waters 2796 Bioseparations Module, Waters 2487 Dual λ Absorbance Detector, XBridge^®^ BEH 200A SEC 3.5 μm 7.8 × 150 mm column, Waters Corporation). The solvent system used was 1× PBS at a 0.4 mL/min flow rate, and the UV-Detector was adjusted to 220 and 280 nm. The analysis of MW and purity of DFO-conjugated M16Ab were performed using Agilent 2100 Bioanalyzer (Agilent High Sensitivity Protein 230 Kit) using the manufacturer’s protocol as previously reported [29].

### 2.9. Radiolabeling with ^89^Zr

^89^Zr in oxalic acid was produced by Saskatchewan Center of Cyclotron Sciences (University of Saskatchewan) as previously reported [30]. Radiolabeling experiments were performed as previously reported [30]. Briefly, 1 M HEPES pH 7.4 (Fisher Scientific) was added to ^89^Zr in oxalic acid followed by dropwise addition of 2 M NaCO_3_ (pH11) while measuring the pH using Hydrion pH paper (range, 5.0–9.0) (Sigma-Aldrich) until the oxalic acid was neutralized (pH 7). DFO-conjugated MUC16 antibody (800 µg) was then added to the ^89^Zr solution at a specific activity of (0.5 MBq/µg) for a total activity of 400 MBq. The reaction mixture was incubated at 37 °C on a shaker at 650 RPM for 90 min. The radiolabeling efficiency was determined by SEC-HPLC using mobile phase and column as described above for the DFO conjugated antibody. Radiochemical purity was also confirmed after spotting a small aliquot (0.5 µL) on a strip of instant thin-layer chromatography silica gel-impregnated paper (iTLC-SG, Agilent Technologies, Santa Clara, CA, USA) using a mobile phase of 50 mM of sodium citrate (pH 5.2).

### 2.10. Stability of ^89^Zr-MUC-16 Ab1

The stability of ^89^Zr-MUC-16 Ab 1 at 37 °C was determined by analyzing aliquots of the radiolabeled antibody using iTLC. The stability of ^89^Zr-M16Ab was evaluated in 1X PBS solution and human plasma at 37 °C for five days (n = 3). ^89^Zr-M16Ab was added to the 1X PBS solution and human plasma to a 15 MBq/mL final concentration. An aliquot of ^89^Zr-M16Ab (triplicates) was drawn every 24 h for five days and analyzed for radiochemical purity by iTLC as described above.

### 2.11. MicroPET/CT Imaging and Biodistribution

Female NOD-SCID mice with MUC16-positive OVCAR3 and SW1990 and MUC16-negative SKOV3 xenografts were injected via a tail vein with 12 ± 1 MBq (21–26 μg) ^89^Zr-DFO-M16Ab. CD-1 nude mice bearing SW1990 xenografts were injected intravenously with 12 ± 1 MBq (21–26 μg) ^89^Zr-DFO-M16Ab. PET/CT imaging was performed at different time points of 24, 48, 72, 96, and 120 h post injection using the Vector4CT scanner (MILabs, Utrecht, The Netherlands). PET scans were acquired in a list-mode data format with a high-energy ultra-high resolution (HE-UHR-1.0 mm) mouse/rat pinhole collimator. Images were constructed using PMOD 3.8 software (PMOD, Switzerland). Mice were sacrificed 24 and 120 h post injection, and major organs and blood were harvested and collected in tubes and then weighed. The radioactivity in all organs and blood was measured using an automated gamma counter (Wallac Wizard 1480, PerkinElmer, Waltham, MA, USA) and expressed as injected radioactive activity per gram (% IA/g).

### 2.12. Pharmacokinetics

Healthy male CD-1 nude mice and mice bearing MUC16-positive SW1990 xenografts (n = 4 per group) were injected via a tail vein with 5–6 MBq of ^89^Zr-DFO-M16Ab (~10–12 μg of M16Ab). Blood samples were collected from the saphenous vein into heparinized capillary tubes at different time points (5 min–5 days). Radioactivity in blood samples was measured using a gamma counter and expressed as % injected activity/mL (% IA/mL). Pharmacokinetic parameters, including the distribution and elimination half-lives (t_1/2_α and t_1/2β_), volume of distribution at steady-state (Vss), clearance (CL), and volume of the central compartment (V_1_), were calculated by fitting the blood radioactivity versus time curve to a two-compartment model with i.v. bolus input.

### 2.13. Statistical Analysis

All data were expressed as the mean ± S.D. of at least three independent experiments. A two-tailed Student’s *t*-test or analysis of variance (ANOVA) with Bonferoni post hoc test were used to assess the statistical significance between the groups. All graphs were prepared and analyzed using GraphPad Prism (version 9; GraphPad, La Jolla, CA, USA).

## 3. Results

### 3.1. Generation and In Vitro Characterization of M16Ab Antibody

**Figure 1:**
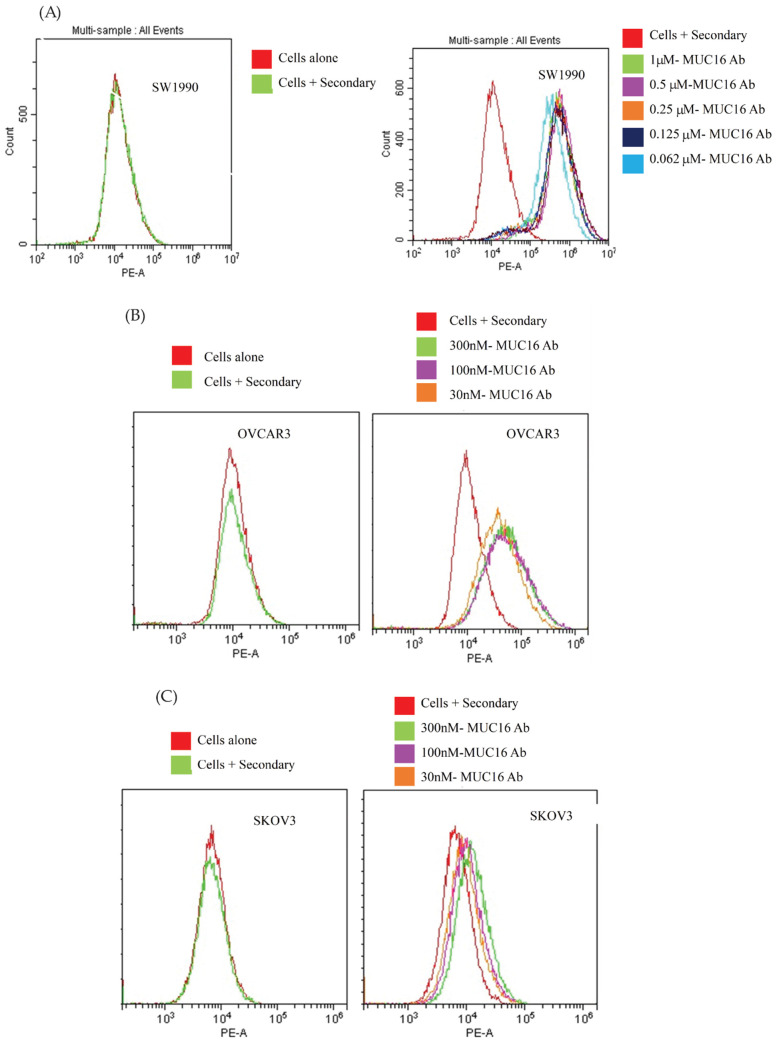
In vitro flow cytometry binding assay results of M16Ab antibody binding on live cells. Different concentrations of M16Ab were incubated with (**A**) MUC16-expressing SW1990 cells, (**B**) MUC16-expressing OVCAR3 cells, and (**C**) MUC-16 negative SKOV3 cells. Dose-dependent binding was observed in both MUC16-expressing cells (**A**,**B**) and not in (**C**) SKOV3 negative-control cell line. Data were analyzed by CytExpert software version 10.

We selected the anti-MUC16 antibody (M16Ab) after phage library panning using a human naïve Fab phage library. Clonal phage ELISA confirmed the specific binding of selected M16Ab Fab-phage to SEA11-12-Fc protein and not to negative-control Fc fusion proteins (Supplementary Figure S2). The full-length IgG1 version of M16Ab was expressed in human-origin Expi293F cells at a yield of 5 mg/L. The bioanalyzer and HPLC confirmed the purity and integrity of the purified M16Ab (Supplementary Figure S3A,B). In vitro binding of M16Ab to high MUC-16-expressing SW1990, OVCAR3, and negative MUC-16 SKOV3 cell lines was determined using flow cytometry. The results showed dose-dependent specific binding with MUC16-positive SW1990 and OVCAR3 and no specific binding was observed with MUC-16-negative SKOV3 cells (Figure 1).

### 3.2. Conjugation of M16Ab and Quality Control of Immunoconjugate

**Figure 2:**
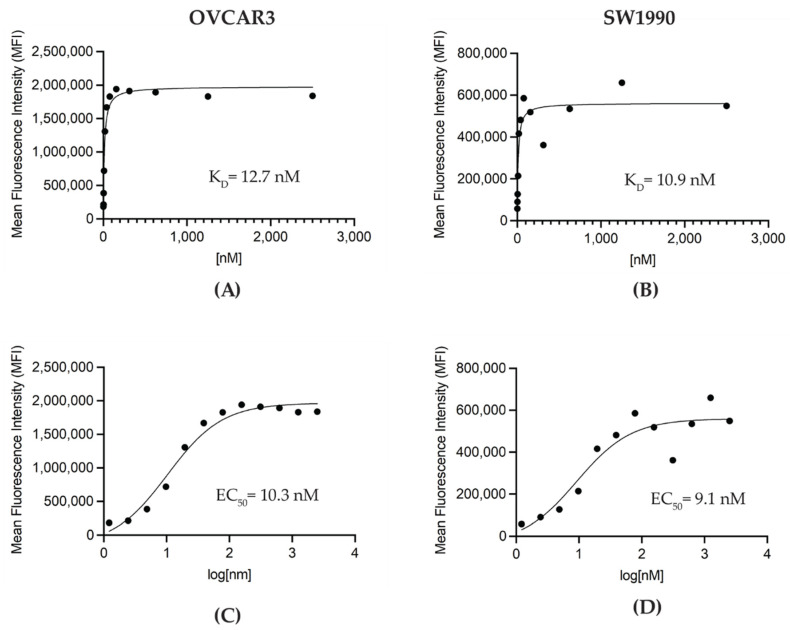
In vitro flow cytometry binding assay. MUC16-expressing OVCAR3 and SW1990 cells were titrated with decreasing concentrations of DFO-M16Ab and analyzed by flow cytometry. (**A**,**C**) OVCAR3 cells, (**B**,**D**) SW1990 cells.

The conjugation of p-SCN-Bn-DFO to M16Ab resulted in >98% pure immunoconjugate with <2% aggregates as evaluated by HPLC (Supplementary Figure S3C). Bioanalyzer was used to characterize the size and purity of DFO-conjugated M16Ab. Bioanalyzer showed that M16Ab and DFO−M16Ab were >91% and >96% pure with molecular weights of 171.3 and 174.6 kDa, respectively (Supplementary Figure S3A,B). Antibodies were conjugated with an average of two DFO molecules per antibody when the ratio of a chelator to the antibody used in the conjugation reaction was 10:1. Like the bioanalyzer results, HPLC profiles were identical between conjugated and unconjugated M16Ab, showing >95% purity. Saturation binding of DFO-M16Ab on MUC16-expressing cells was performed using flow cytometry to study the effect of DFO conjugation on M16Ab binding. Mean fluorescence intensity (MFI) was plotted against concentration to calculate the binding constant K_D_ and EC_50_ values of immunoconjugate for MUC-16 expressing SW1990 and OVCAR3 cells. The estimated K_D_ values for M16Ab and DFO-M16Ab were 10.9 and 12.7 nM, respectively (Figure 2A,B). The estimated EC_50_ values for M16Ab and DFO− M16Ab were 9.1 and 10.3 nM, respectively (Figure 2C,D).

### 3.3. Internalization of M16Ab

**Figure 3:**
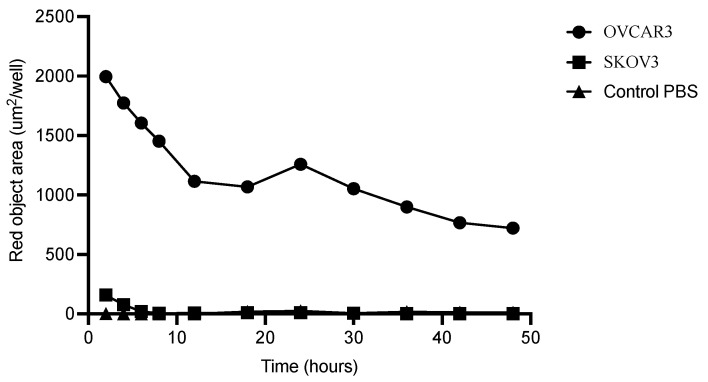
Internalization of MUC16 antibody: MUC16-positive OVCAR3 and MUC16-negative SKOV3 cells were treated with IncuCyte FabFluor-labeled M16Ab antibody (4 μg/mL); HD phase and red fluorescence images (10×) were captured every 2 h for 48 h. All data are shown as a mean of three wells ± SEM.

Rapid internalization of M16Ab was observed in the positive OVCAR3 cells compared to the 13-fold lower internalization observed in the negative SKOV3 cells following two hours of incubation. After 12, 30, and 48 h incubation, M16Ab showed 180-, 351- and 721-fold higher internalization in OVCAR3 cells compared to the negative SKOV3 cells. No internalization was observed in the media control for up to 48 h. Most of M16Ab was internalized in OVCAR3 cells by the first 30 h which showed 1.5-fold higher internalization compared to 48 h (Figure 3).

### 3.4. Radiolabeling and Characterization

The radiolabeling of DFO-M16Ab with ^89^Zr at a specific activity of 0.5 MBq/μg resulted in a radiochemical yield of 393 MBq (>98%) (Supplementary Figure S3C). The stability of ^89^Zr-M16Ab1 was evaluated at different time points by iTLC at 37 °C in 1xPBS and human plasma. In both human plasma and PBS, >97% and 96%, respectively, of ^89^Zr-M16Ab remained intact for 72 h of incubation at 37 °C but decreased slightly after 5 days. Overall, the radioimmunoconjugate was stable in human plasma and PBS after incubation at 37 °C for 5 days (Supplementary Figure S4).

### 3.5. Pharmacokinetics of M16Ab in CD-1 Mice

**Figure 4:**
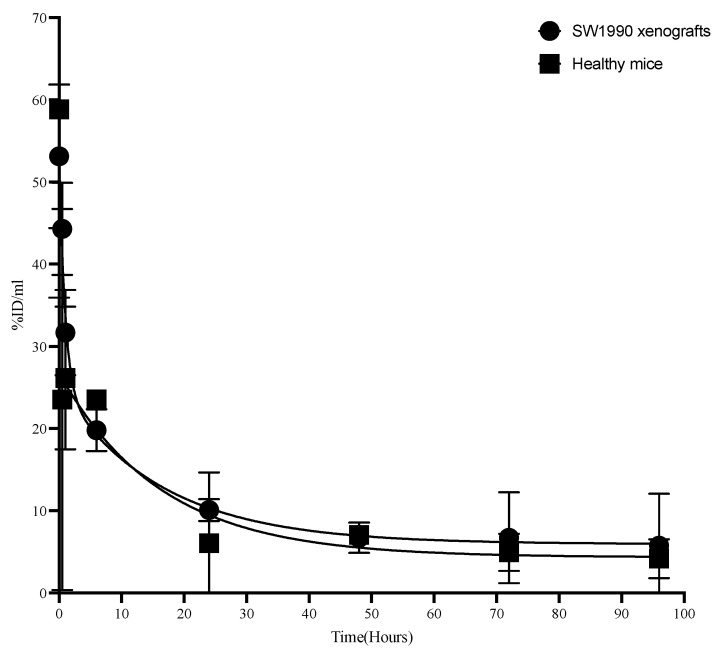
Pharmacokinetic evaluation of ^89^Zr-DFO-M16Ab in normal CD-1 nude mice.^89^Zr-DFO-M16Ab showed a bi-phasic half-life with (distribution) half-life t_1/2α_ of 4.42 h and a slow clearance t_1/2β_ of 99 h. The volume of distribution of the central compartment (V1) was 2.9 mL (116 mL/kg), and the volume of distribution at steady state (Vss) was 7.69 mL (307 mL/kg). The V1 and Vss volumes were 8.1 L and 21.4 L, respectively, in a 70 kg standard adult female. The systemic clearance (CLs) was 0.058 mL/h (2.3 mL/h/kg). The CLs values in mice would correspond to 161 mL/h in a 70 kg adult female.

Pharmacokinetics of ^89^Zr-M16Ab were studied in non-tumor-bearing and tumor-bearing CD-1 nude mice to understand if antigen shedding would influence the binding and hence the kinetics of the antibody. ^89^Zr-M16Ab showed a bi-phasic half-life with distribution half-life t_1/2α_ of 5.8 ± 2.6 and 2.64 ± 1.3 and a moderate clearance t_1/2β_ of 92.4 h ± 15.2 and 63.58 h ± 19.5 (Table 1) in mice bearing MUC16-expressing tumors and healthy mice, respectively (Figure 4).

### 3.6. Biodistribution and MicroPET/CT Imaging in Tumor-Bearing Mice

**Figure 5:**
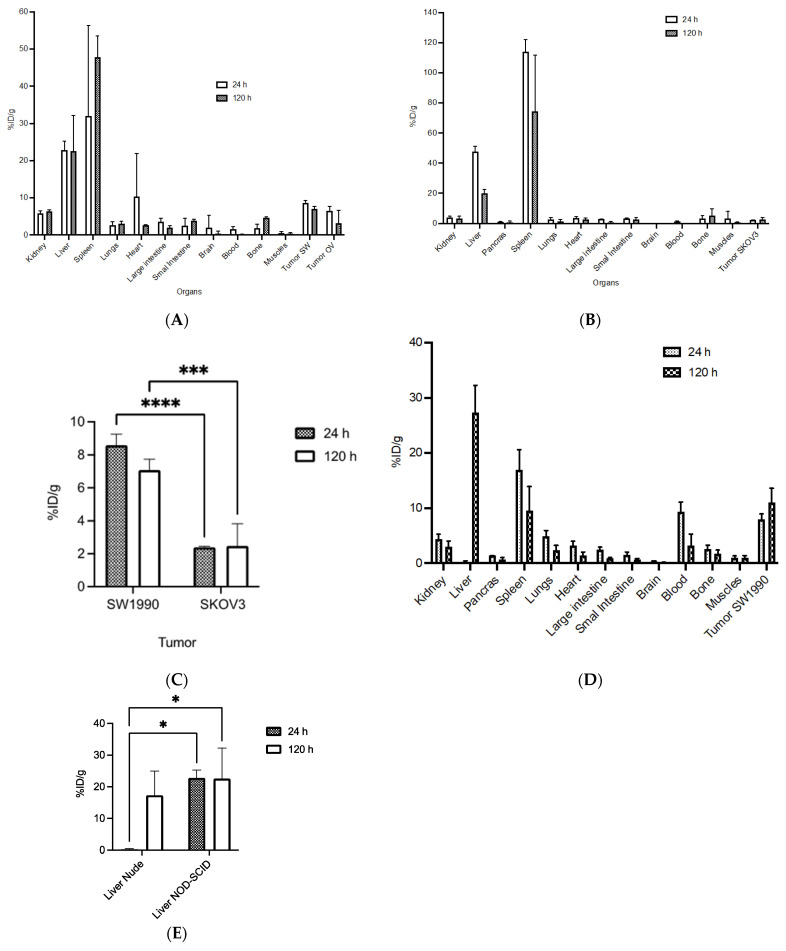
Biodistribution of ^89^Zr-DFO-M16Ab in MUC16-positive SW1990, OVCAR3, and negative-control SKOV3 xenografts at 24 h and 120 h post injection. (**A**,**B**) NOD-SCID mice bearing xenografts were injected intravenously with 10 MBq 20 μg of ^89^Zr-DFO-M16Ab followed by biodistribution studies. (**C**) Uptake in SW1990 and OVCAR3 tumors was significantly higher than in negative-control SKOV3 at 24 h (*** *p* < 0.0001) and 120 h (**** *p* < 0.0008) post injection. (**D**) CD-1 nude mice bearing MUC16-expressing SW1990 xenografts were injected intravenously with 10 MBq 20 μg of ^89^Zr-DFO-M16Ab followed by biodistribution studies. (**E**) Liver uptake in NOD-SCID mice was significantly higher than in CD-1 nude mice at 24 h and 120 h (* *p* < 0.01) post injection.

^89^Zr-M16Ab biodistribution was determined at 24 and 120 h p.i. in NOD-SCID mice bearing xenografts with high MUC16 expression (SW1990, OVCAR3) and a control negative for MUC16 expression (SKOV3) (Figure 5A,B). Additionally, the biodistribution was measured at the same time points p.i. in CD-1 nude mice bearing SW1990 xenograft. In NOD-SCID mice, tumor uptake in %IA/g was slightly higher in SW1990 (8.6 ± 0.6% IA/g) than for OVCAR3 at 24 h p.i. (6.5 ± 1.2% IA/g) and was not significant. This uptake decreased to (7.1 ± 0.7% IA/g) and (3.17 ± 3.5% IA/g), respectively, at 120 h p.i. Negative-control xenograft had significantly lower tumor uptake at 24 h p.i. (2.4 ± 0.1% IA/g) and 120 h p.i. (2.5 ± 1.3% IA/g) (Figure 5C). There was a significantly high uptake in the spleen followed by the liver (spleen: 28.6 ± 18.39% IA/g; liver: 22.8 ± 2.5% IA/g) of the labeled antibody at 24 h p.i. However, this increased to (47.8 ± 5.6% IA/g) for the spleen and remained the same (22.5 ± 9.6% IA/g) for the liver at 120 h p.i. There was a high uptake observed in the heart at the early time point of 24 h of (7.67 ± 8.9% IA/g) due to the blood pool, however, this was decreased over time to (2.6 ± 0.19% IA/g) at the 120 h p.i. The tumor-to-blood ratio for SW1990 and OVCAR3 was 5.7 and 4.3, respectively, at 24 h p.i. However, at 120 h p.i., the ratio increased to 46.6 and 21, respectively. The tumor-to-muscle ratio at 24 h was 16- and 10-fold higher for SW1990 and OVCAR3, respectively, compared with negative-control SKOV3, and at 120 h, the ratio was 10- and 11-fold higher.

In CD-1 nude mice bearing high MUC16-expressing SW1990 xenograft, tumor uptake increased over time from (7.9 ± 1.0% IA/g) at 24 h p.i. to 11.6 ± 2.1% IA/g at 120 h p.i. (Figure 5D). There was almost no uptake in the liver (0.25 ± 0.21% IA/g) at 24 h p.i. but this increased to (17.3 ± 7.6% IA/g) at 120 h p.i. The spleen had an uptake of (16.9 ± 3.6% IA/g) at 24 h p.i. However, this decreased to (12.1 ± 0.54% IA/g) at 120 h p.i. This showed that the uptake in the spleen and liver was significantly lower than what was observed in the NOD-SCID mice (Figure 5E). At the early time point of 24 h, the lungs and heart uptake were observed to be (lungs: 4.9 ± 1.0% IA/g; heart: 3.2 ± 0.8% IA/g) but this uptake was decreased to (lungs: 0.8 ± 0.2% IA/g; heart: 0.4 ± 0.1% IA/g) at 120 h p.i. The highest tumor-to-blood ratio was 3.3 at 120 h p.i. The tumor-to muscle ratio was 8.2 and 7.6 at 24 and 120 h, respectively.

**Figure 6:**
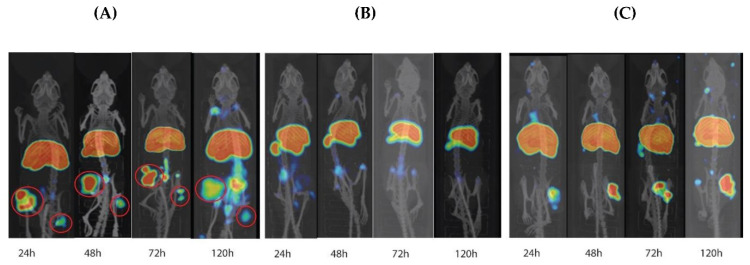
PET/CT imaging and image analyses in mice xenografts. (**A**) Maximum intensity projection (MIP) PET/CT images of a representative NOD-SCID mouse bearing MUC16-positive SW1990 and OVCAR3, SW1990, and OVCAR3 xenografts are in the same mouse on the left and right thigh of the hind leg, respectively. Red circles highlight the tumor (**B**) Maximum intensity projection (MIP) PET/CT images of a mouse bearing negative-control SKOV3 xenografts at different time points post-^89^Zr-DFO-M16Ab injection. (**C**) Maximum intensity projection (MIP) PET/CT images of a representative CD-1 nude mouse bearing MUC16-positive SW1990 xenografts on the right flank at different time points post-^89^Zr-DFO-M16Ab injection.

PET imaging showed high tumor uptake in MUC16-positive xenografts as seen in the maximum intensity projection images of the NOD-SCID mice at 24–120 h p.i. (Figure 6A). There was no significant difference (*p* > 0.9999) in uptake between the two MUC16-expressing xenografts at 24 h or 120 h p.i. Tumor uptake was higher at 24 h p.i. and then decreased over time. Up to 120 h p.i., there was no uptake observed in the negative-control xenografts on microPET (Figure 6B). Unlike the observation in NOD-SCID mice, the maximum intensity projection images of the CD-1 nude mice showed increased accumulation of ^89^Zr-M16Ab in MUC16-expressing SW1990 tumors overtime in which, the highest tumor uptake was observed at 120 h p.i. (Figure 6C).

## 4. Discussion

A serum level of CA125 is the most extensively used biomarker for EOC. However, the sensitivity and specificity of detection are 67.39% and 86.79%, respectively [31]. A CA125 serum concentration of ≥35 U/mL is suggestive of potential malignancies/recurrence, with a 47% elevation in EOC early stage and an 80–90% elevation in the advanced stage [32]. However, many benign conditions also have high levels of CA125 [33]. Non-invasive molecular imaging using positron emission tomography (PET) offers many obvious advantages over ex vivo methods such as immunohistochemistry (IHC) or serum CA125 levels [34,35]. Moreover, immunoPET targeting CA125 expression is useful in PDAC where serum CA125 levels are not currently used for diagnosing pancreatic cancer.

For cancer therapy, unlike humanized antibodies, fully human antibodies are the most desirable format for clinical applications as they do not contain any murine sequences and therefore eliminate the concerns for immunogenicity [36]. Here, we described the development and evaluation of M16Ab, a fully human antibody, to target the MUC16/CA125 antigen overexpressed in EOC and PDAC. This study is the first report of a fully human monoclonal anti-CA125/MUC16 antibody for PET imaging of CA125/MUC16-expressing cancers with potential for further development as radiopharmaceuticals for targeted therapy.

The choice of PET isotope with a long physical half-life that matches an antibody’s relatively slow pharmacokinetics distribution is crucial for constructing an effective antibody-based nuclear imaging probe. ^89^Zr has become a popular choice for preclinical and clinical immunoPET imaging owing to its desirable decay properties. Its sufficiently long physical half-life (78.4 h) and its emission of positrons result in higher-resolution imaging [37]. Therefore, M16Ab was conjugated with *p*-SCN-Bn-DFO, which resulted in a highly pure immunoconjugate with low nanomolar affinity (11 nM), as shown by bioanalyzer/HPLC and flow cytometry. DFO-M16Ab was efficiently labeled with ^89^Zr and was stable for up to 5 days. The in vivo specificity and tumor uptake of ^89^Zr-M16Ab were assessed using microPET and biodistribution studies. ^89^Zr-M16Ab showed early high uptake in MUC16-positive tumor xenografts in the NOD-SCID mice. The highest tumor uptake of (8.6% IA/g) was observed at 24 h p.i., and this uptake decreased over time to (7.1% IA/g) by the end of imaging at 120 h p.i. Fast clearance of ^89^Zr-M16Ab from blood (2% IA/g) was observed as early as 24 h p.i, and increasing spleen and liver uptake were observed up to 120 h p.i. These results were in accordance with the findings from mouse studies showing that mice with SCID mutation could substantially reduce the tumor uptake, due to low endogenous IgG levels, through rapid clearance of the radiolabeled antibody from the blood into the non-target organs such as the spleen, liver, and bones [38,39]. In contrast, these observations were reversed in our imaging and biodistribution results of CD-1 nude mice. The tumor uptake increased over time from 7.9% IA/g at 24 h p.i. to ~12% IA/g at 120 h p.i. The radiotracer uptake in the spleen and liver was significantly lower than what was observed in the NOD-SCID mice. This may be attributed to the ~5-fold slower blood clearance of ^89^Zr-M16Ab from blood in nude mice compared to NOD-SCID. The specificity of tumor uptake was validated by imaging and biodistribution studies of mice bearing MUC16-negative SKOV3 xenografts.

In comparison to other antibody-based immunoPET probes developed against CA125/MUC16, we developed a fully human monoclonal antibody for the non-invasive imaging of MUC16-expressing cancers. Unlike the ^89^Zr-DFO-mAb-B43.13 by Sharma et al. [24], we efficiently labeled our antibody with ^89^Zr at a high specific activity of (0.5 MBq/1 μg). This allowed us to administer a minimal dose of ^89^Zr-M16Ab of (10–12 MBq and 20–24 μg) compared with their (10–12 MBq and 40–50 μg) dose and to get highly specific tumor targeting while minimizing the amount of radiotracer residing in off-target organs such as the liver. Although they had higher tumor uptake in OVCAR3 xenografts at 12 h p.i, the uptake of our radiotracer was higher at 24 h p.i. with almost no uptake observed in the liver resulting in a high tumor-to-liver ratio of 32 at 24 h. We demonstrated these results not only in EOC but also in PDAC models.

One concern about MUC16 as a therapeutic/imaging target is that the cleavage and shedding of the extracellular domain of MUC16 in serum or in the peritoneal fluid of patients may reduce or inhibit the accumulation of M16Ab in the tumor and obscure the visualization of small lesions [40]. We evaluated the therapeutic potential of M16Ab by conducting a pharmacokinetic (PK) study to compare the PK behavior of M16Ab in mice with large MUC-16-overexpressing xenografts vs. healthy mice. The PK profile of radiolabeled M16Ab was similar for both models with a fast distribution half-life t_1/2α_ of 5.8 h and a moderate clearance t_1/2β_ of 92.4 h. Additionally, our antibody showed a 13-fold higher internalization in MUC16-expressing OVCAR3 cells compared to negative-control SKOV3 cells in the first 2 h of incubation, and this internalization was 721-fold higher by the end of 48 h incubation, indicating the therapeutic potential of M16Ab via antibody-drug conjugates or the delivery of highly toxic radiation dose to the tumor site. Due to its slow internalization, MUC16 is considered a poor therapeutic target for the immunoconjugates or ADCs that act inside tumor cells [41]. However, its overexpression on the surface of cancer cells makes it a desirable therapeutic biomarker for targeted radioimmunotherapy (T-RIT) using high-energy short-range radioisotopes, where internalization is not a limiting factor. Immunoconjugates with high specificity and affinity to MUC16 can deliver a tumoricidal radiation dose to cancer cells [42].

Although M16Ab is a promising theranostic probe, it has some limitations. The expression of full-length IgG in mammalian cells results in a low yield of 5 mg/L. Stabilizing complementarity-determining region (CDR) loops with site-directed mutagenesis has previously been applied for improving antibody yields. M16Ab may benefit from further optimization of CDR loops. In this study, we have demonstrated that by conjugating a high contrast agent such as ^89^Zr with our M16Ab, we could effectively and specifically deliver this contrast agent into MUC16-expressing tumors while restricting the distribution to normal organs, therefore, reducing the potential for off-target toxicities. The high-resolution images and reduced background non-target (except for the liver and spleen) uptake of the ^89^Zr-M16Ab probe would allow for better visualization of metastatic lesions. We intend to evaluate the therapeutic potential of our antibody by radiolabeling with potent therapeutic isotopes. The potential/effectiveness of such a radioimmunoconjugate will be evaluated in animal models that closely resemble EOC and PDA cancers in patients by using patient-derived xenografts (PDX) models.

## 5. Conclusions

In this study, we have described the development and the preclinical evaluation of ^89^Zr-DFO-M16Ab as a PET imaging probe of MUC16-expressing cancers. Unlike the murine-origin monoclonal antibodies, our anti-MUC16 antibody is fully human and is expected to be less immunogenic, which is essential for translation into effective drugs. This will enable early detection of EOC and PDAC and provides a reliable prognosis tool for monitoring the progression of the disease. The specificity of targeting and the high tumor uptake make our developed anti-MUC16 antibody a great contrast agent for non-invasive imaging and show potential for further development as radiopharmaceuticals for targeted therapy of MUC16-expressing cancers.

## Figures and Tables

**Table 1:** Pharmacokinetics of ^89^Zr-M16Ab in normal and tumor-bearing mice bearing pancreatic SW1990 MUC-16 positive tumor.

	AUC (%IA.h/mL)	t_1/2α_ (h)	t_1/2ß_ (h)	CL (mL)	Vss (mL/h × 10^−2^)
Tumor-bearing mice	979.0 ± 197.6	5.8 ± 2.6	92.4 ± 15.2	0.11 ± 0.01	9.5 ± 2.4
Normal mice	850.0 ± 245	2.64 ± 1.3	63.58 ± 19.5	0.14 ± 0.04	11.1 ± 6.1

## Data Availability

Not applicable.

## Supplementary Materials

The following supporting information can be downloaded at: https://www.mdpi.com/article/10.3390/pharmaceutics14122824/s1. Figure S1: SEA11-12 domain of MUC16 cloned into pMUFV-01-Fc a custom lentiviral vector; Figure S2: Clone ELISA illustrating binding specificity of M16-Fab tested with SEA 11–12 domain recombinant protein (blue) and Fc protein as negative control (orange). All 48 clones displayed the same signal and were the same sequence; Figure S3: (A,B) Bioanalyzer ladder (A) and chromatograms (B) of ladder, M16Ab and DFO-M16Ab. (C) Representative size exclusion (SEC) HPLC chromatograms showing stability of M16Ab, DFO-M16Ab and 89Zr-DFO-M16Ab. UV channel (280) and radiometric channels are shown; Figure S4: Stability studies of 89Zr-DFO-M16Ab at room in human plasma and PBS at 37 °C.
