# Supplementary material for: Engineering of a Fully Human Anti-MUC-16 Antibody and Evaluation as a PET Imaging Agent
Source: Pharmaceutics. 2022 Dec 16;14(12):2824. doi: 10.3390/pharmaceutics14122824 (PMC9785263; doi:10.3390/pharmaceutics14122824)
Supplement: Supplementary file 1 [file pharmaceutics-14-02824-s001.zip › pharmaceutics-2015341-supplementary.pdf]

Supplementary Information

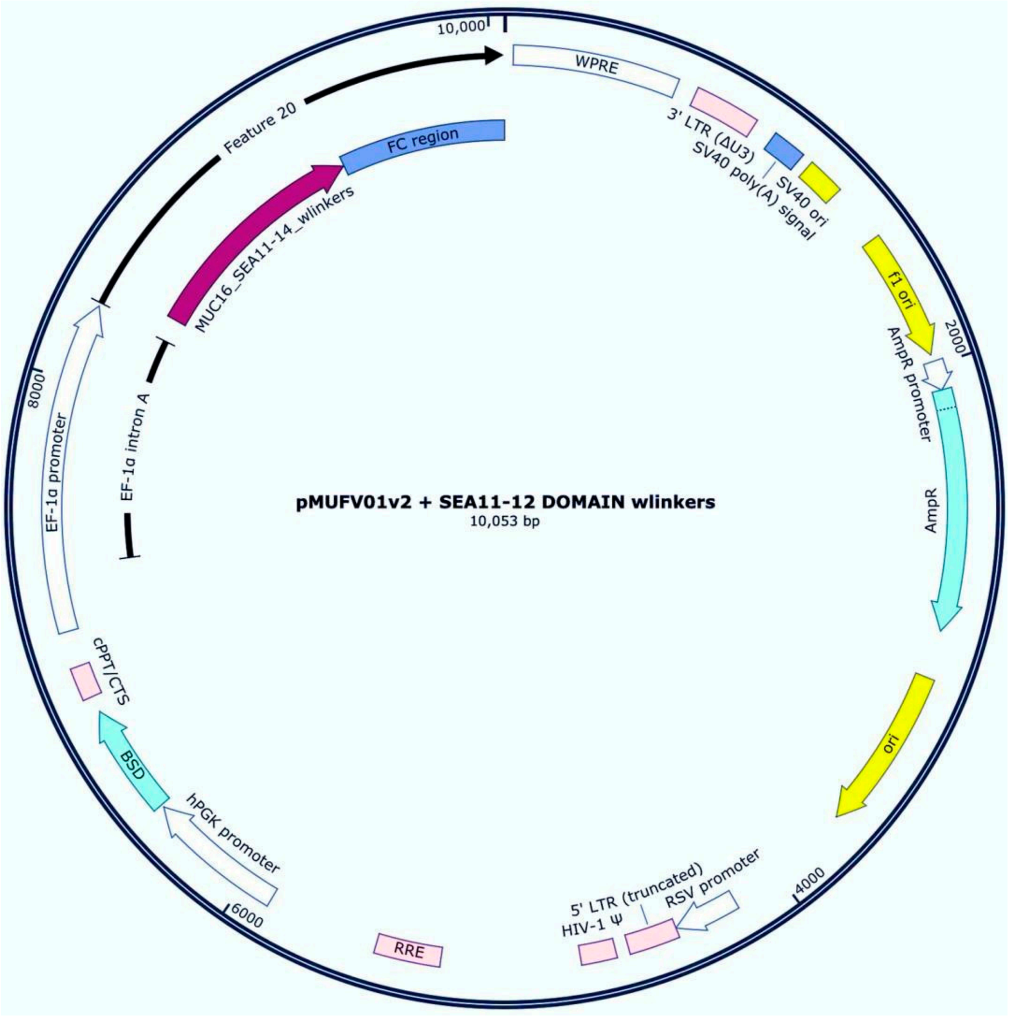

Supplementary Figure S1: SEA11-12 domain of MUC16 cloned into pMUFV-01-Fc a custom lentiviral vector

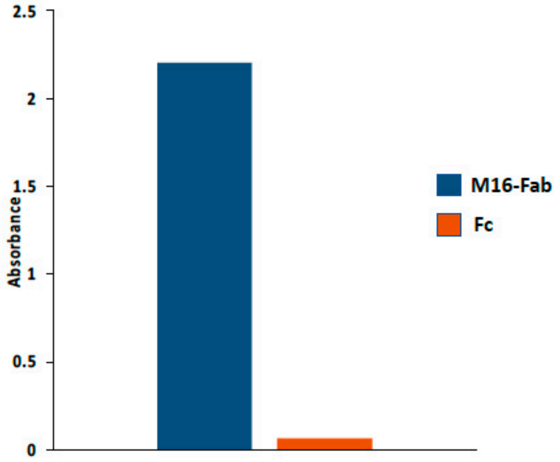

Supplementary Figure S 2: Clone ELISA illustrating binding specificity of M16-Fab tested with SEA 11-12 domain recombinant protein (blue) and Fc protein as negative control (orange). All 48 clones displayed the same signal and were the same sequence.

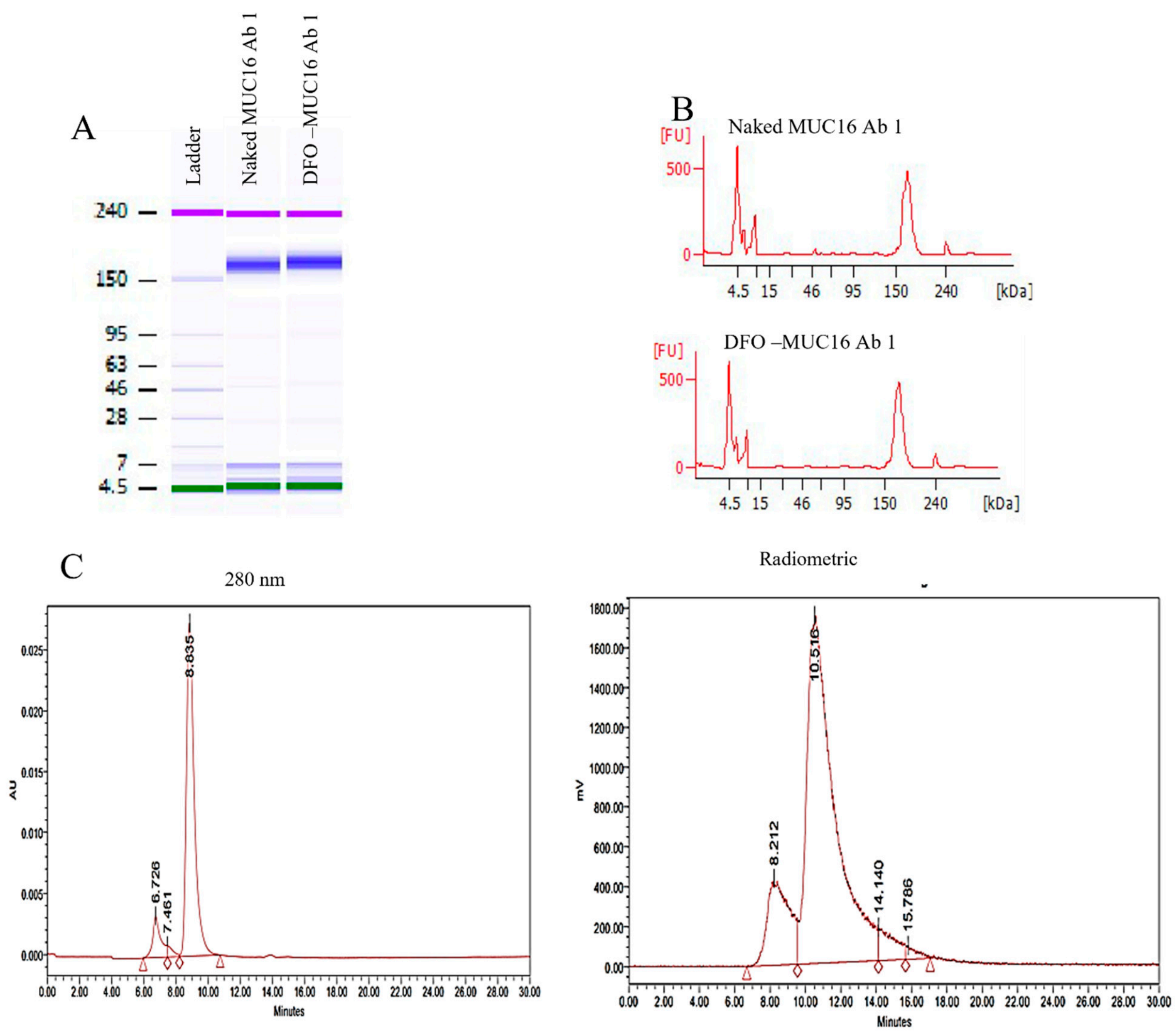

Supplementary Figure S3: (A and B) Bioanalyzer ladder (A) and chromatograms (B) of ladder, M16Ab and DFO-M16Ab. **(C)** Representative size exclusion (SEC) HPLC chromatograms showing stability of M16Ab, DFO-M16Ab and ^{89}Zr-DFO-M16Ab. UV channel (280) and radiometric channels are shown.

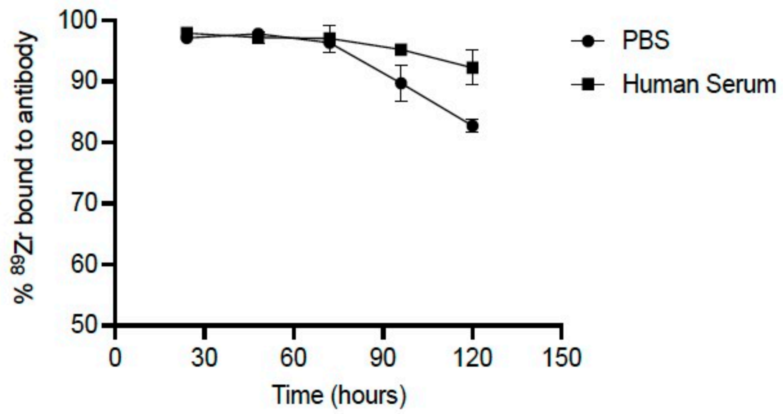

Supplementary Figure S4: Stability studies of 89Zr-DFO-M16Ab at room in human plasma and PBS at 37°C
